# Automated brain atrophy quantification from clinical MRI predicts early neurological deterioration in anterior choroidal artery territory infarction

**DOI:** 10.3389/fnins.2025.1714159

**Published:** 2025-12-18

**Authors:** Weiwei Gao, Shouyue Jin, Zhimin Xiao, Lixue Wang, Jianzhong Lin, Xingyu Chen, Renjing Zhu, Aihuan Zhang

**Affiliations:** 1 Department of Neurology, Zhongshan Hospital of Xiamen University, School of Medicine, Xiamen University, Xiamen, China; 2 Xiamen Clinical Research Center for Cerebrovascular Diseases, Xiamen, China; 3 Department of Neurology, Jimusaer County People’s Hospital, Xinjiang, China; 4 Department of Magnetic Resonance Imaging, Zhongshan Hospital of Xiamen University, School of Medicine, Xiamen University, Xiamen, China; 5 Department of Ophthalmology, Zhongshan Hospital of Xiamen University, School of Medicine, Xiamen University, Xiamen, China

**Keywords:** anterior choroidal artery, infarction, early neurological deterioration, brain atrophy quantification, automated neuroimaging

## Abstract

**Background:**

Early neurological deterioration (END) occurs in 43%–60% of patients with anterior choroidal artery (AChA) territory infarction. While brain atrophy serves as an imaging biomarker of diminished brain reserve capacity and may influence stroke outcomes, its predictive value for END in AChA infarction remains unclear.

**Methods:**

This dual-center retrospective cohort study consecutively enrolled patients with acute AChA territory infarction admitted to two Chinese stroke centers between September 2018 and September 2024. Clinical T1-weighted images were reconstructed into standardized high-resolution images using the SynthSR deep learning algorithm, followed by fully automated brain tissue segmentation via AssemblyNet. We calculated gray matter fraction (GMF), white matter fraction (WMF), brain parenchymal fraction (BPF), and cerebrospinal fluid fraction (CSFF) to quantify brain atrophy severity. Multivariable logistic regression and restricted cubic spline (RCS) analyses were employed to assess associations between brain atrophy metrics and END.

**Results:**

Among 206 enrolled patients, 78 (37.86%) developed END. Patients with END demonstrated significantly greater brain atrophy: GMF (*P* < 0.001), WMF (*P* < 0.001), and BPF (*P* < 0.001) were all significantly reduced, while CSFF was correspondingly elevated (*P* < 0.001). In fully adjusted models, each 0.01-unit increase in WMF was associated with a 58% reduction in END risk (*P* < 0.001); each 0.01-unit increase in BPF corresponded to a 32% risk reduction (*P* = 0.002); and each 0.01-unit increase in CSFF was associated with a 52% increase in risk (*P* < 0.001). Quartile analysis confirmed dose-response relationships: the highest quartiles of WMF and BPF were associated with 91% and 74% reductions in END risk, respectively, while the highest CSFF quartile conferred a 6.2-fold increased risk. RCS analysis confirmed significant linear dose-response relationships between both BPF and CSFF with END (both *P*-non-linear > 0.05).

**Conclusion:**

Automated brain atrophy quantification based on routine clinical MRI can independently predict END risk in patients with AChA infarction, providing a feasible imaging biomarker for this high-risk stroke population and facilitating early risk stratification and treatment optimization.

## 1 Introduction

Acute ischemic stroke remains one of the leading causes of death and disability worldwide (Feigin et al., 2023). Among various stroke subtypes, anterior choroidal artery (AChA) territory infarction has garnered particular clinical attention due to its unique vascular anatomical characteristics and exceptionally high incidence of early neurological deterioration (END). The AChA originates from the supraclinoid segment of the internal carotid artery and supplies critical functional regions including the posterior limb of the internal capsule, cerebral peduncle, lateral thalamus, lateral geniculate body, and medial temporal structures (Antoniello et al., 2023; Herman et al., 1966). Consequently, AChA territory infarction frequently results in severe motor, sensory, and visual dysfunction. Of greater concern, 43%–60% of patients with AChA territory infarction develop END, which significantly compromises functional outcomes and long-term prognosis (Cheng et al., 2020; Derflinger et al., 2015; Zhao et al., 2023).

Recent investigations have established strong associations between neuroimaging markers of brain frailty—including brain atrophy, lacunar infarcts, white matter hyperintensities, and enlarged perivascular spaces—and post-stroke functional outcomes (Bu et al., 2021; Cheng et al., 2025). Among these, brain atrophy serves as a crucial imaging biomarker of diminished brain reserve capacity and is considered an objective indicator of biological brain aging (Pedraza et al., 2020). Individuals with greater brain reserve demonstrate enhanced resilience to acute brain injury, superior modulation of post-injury functional recovery processes, and improved compensatory capacity for infarct lesions of given volumes (Luijten et al., 2021). However, clinical assessment of brain atrophy predominantly relies on visual rating scales, such as the global cortical atrophy scale and medial temporal lobe atrophy scale, which possess inherent limitations in quantitative precision. While volumetric quantification methods can provide more accurate brain atrophy assessment, they face practical challenges in acute stroke clinical settings. The imperative for rapid stroke diagnosis and treatment necessitates expedited MRI scanning protocols that maintain adequate image quality for qualitative assessment but sacrifice the high resolution required for quantitative analysis. High-resolution three-dimensional magnetization-prepared rapid gradient echo (3D-MPRAGE) sequences, essential for precise morphological analysis, require extended acquisition times that limit their applicability in acute clinical practice.

In 2021, Iglesias and colleagues developed SynthSR, a convolutional neural network-based tool capable of reconstructing clinical brain MRI scans of arbitrary orientation, resolution, and contrast into standardized 1-mm isotropic 3D-MPRAGE-style images (Iglesias et al., 2023). Large-scale validation across more than 10,000 multi-center datasets demonstrated that SynthSR-reconstructed images exhibit accuracy and reliability highly consistent with original high-resolution images in morphological analyses. This technology creates an important bridge between real-world stroke care and scientific research, enabling comprehensive utilization and in-depth analysis of the vast heterogeneous imaging datasets generated in clinical practice.

Based on this background, the present study aimed to leverage SynthSR deep learning technology to achieve automated brain atrophy quantification from routine clinical MRI and to systematically investigate associations between quantitative brain atrophy metrics and END risk in patients with AChA territory infarction. We hypothesized that greater brain atrophy severity would be independently associated with increased END risk. These findings would provide important imaging biomarkers and scientific evidence for improving risk stratification and individualized treatment strategies in this high-risk stroke subtype.

## 2 Materials and methods

### 2.1 Study design and patient selection

**FIGURE 1:**
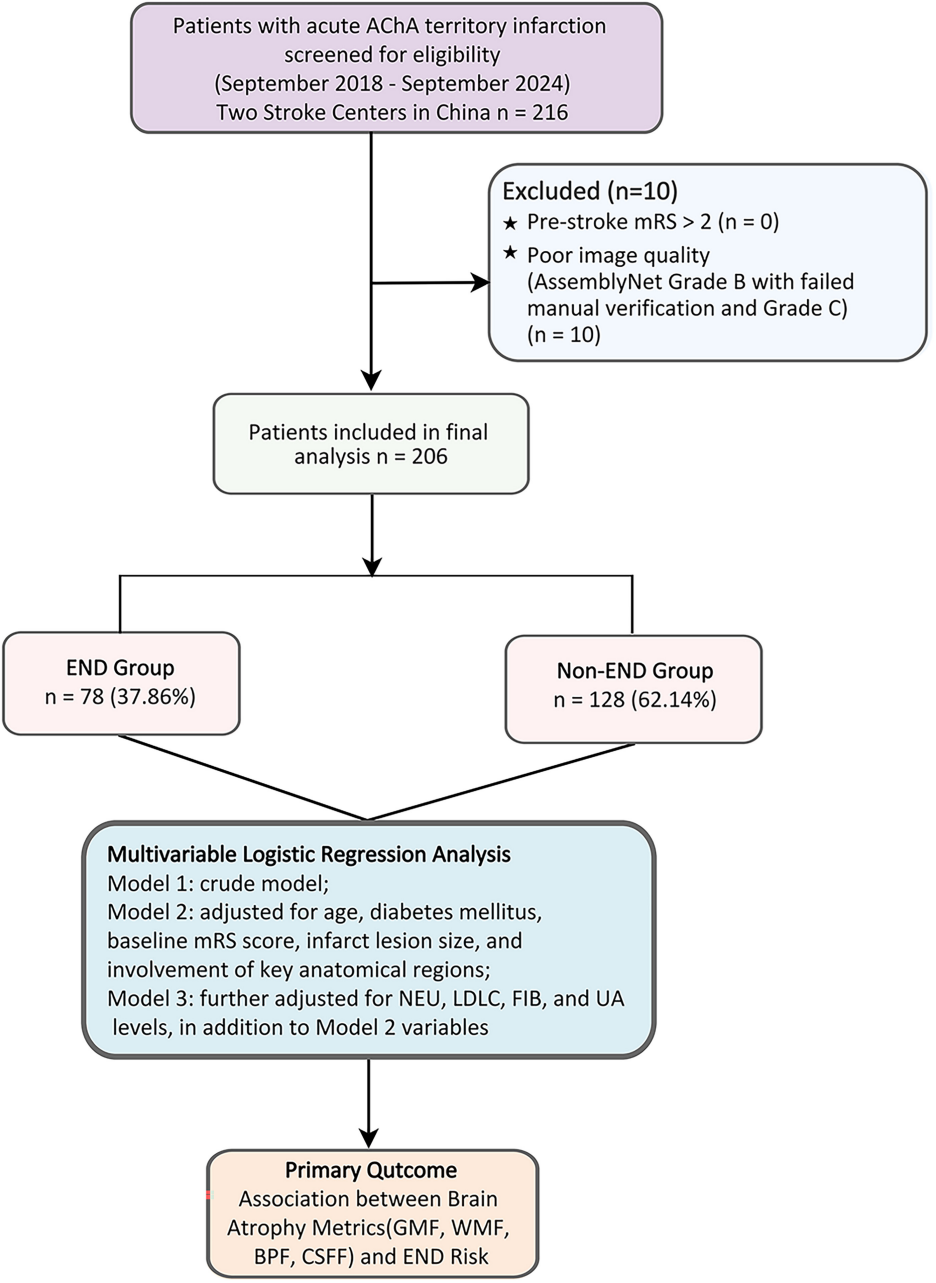
Patient selection and analytical framework. AChA, anterior choroidal artery; BPF, brain parenchymal fraction; CSFF, cerebrospinal fluid fraction; END, early neurological deterioration; FIB, fibrinogen; GMF, gray matter fraction; LDLC, low-density lipoprotein cholesterol; mRS, modified Rankin Scale; NEU, neutrophil count; UA, uric acid; WMF, white matter fraction.

This retrospective cohort study consecutively enrolled patients with AChA territory infarction admitted to two Chinese stroke centers between September 2018 and September 2024. Inclusion criteria comprised: (1) age ≥ 18 years; (2) acute ischemic lesions in the AChA territory confirmed by diffusion-weighted imaging (DWI); and (3) complete clinical data availability. We excluded patients with pre-stroke modified Rankin Scale (mRS) scores > 2 and those with poor image quality precluding accurate analysis. The patient selection process is illustrated in Figure 1.

Radiological diagnosis of AChA territory infarction was established according to the following criteria (Antoniello et al., 2023; Herman et al., 1966): (1) the main body of the lesion located within the probabilistic distribution atlas of the AChA territory^1^; and (2) infarct involvement of ≥1 of the following anatomical structures, with exclusion of concurrent involvement of other arterial territories: posterior two-thirds of the posterior limb of the internal capsule, posterior periventricular region (corona radiata), lateral thalamus, lateral geniculate body, medial globus pallidus, tail of the caudate nucleus, medial temporal lobe, hippocampus and parahippocampal gyrus, and anterior one-third of the cerebral peduncle base.

### 2.2 Data collection

We collected demographic characteristics (age, sex) and cerebrovascular risk factors (smoking history, alcohol consumption, hypertension, diabetes mellitus, hyperlipidemia, atrial fibrillation, coronary artery disease, and prior stroke/transient ischemic attack). Neurological deficit severity was assessed using the National Institutes of Health Stroke Scale (NIHSS) and mRS scores, both administered by certified, trained neurologists. Imaging characteristics included infarct lesion size and specific anatomical regions involved. Treatment-related variables encompassed intravenous thrombolysis and antiplatelet therapy strategies (no antiplatelet therapy, single, or dual antiplatelet therapy). Fasting venous blood samples were obtained on the morning following admission for laboratory analysis, including: complete blood count parameters (white blood cell count, neutrophil count, lymphocyte count, monocyte count, red blood cell count, hemoglobin concentration, platelet count), biochemical markers (total protein, albumin, alanine aminotransferase, aspartate aminotransferase, triglycerides, total cholesterol, low-density lipoprotein cholesterol, serum creatinine, uric acid), fibrinogen, and fasting glucose.

### 2.3 Image acquisition

Brain MRI data were acquired using Siemens Healthcare (Erlangen, Germany) 1.5T or 3.0T magnetic resonance imaging systems at both participating centers. All patients underwent standardized clinical scanning protocols including T1-weighted imaging (T1WI), T2-weighted imaging (T2WI), fluid-attenuated inversion recovery (FLAIR), and diffusion-weighted imaging (DWI). Following institutional radiology protocols, T1WI was performed using gradient echo sequences with the following typical parameters (based on standard protocols and available DICOM metadata): repetition time (TR) 250 ms, echo time (TE) 2.48 ms, flip angle 70°, acquisition matrix 256 × 256, slice thickness 5.0 mm, and 28 slices. Given the retrospective nature of this study, minor variations in actual acquisition parameters may have occurred across the study period and individual scans.

To address the inherent heterogeneity arising from multi-scanner, multi-field-strength data acquisition, we employed the SynthSR deep learning tool (v1.0) (Iglesias et al., 2023) to standardize all T1WI. SynthSR reconstructs clinical scans of arbitrary field strength, resolution, and contrast into uniform 1-mm isotropic MPRAGE-style images, automatically performing contrast standardization, bias field correction, and signal-to-noise ratio optimization via convolutional neural networks. This tool has been extensively validated across more than 10,000 multi-center datasets, demonstrating robust performance in harmonizing heterogeneous clinical images.

### 2.4 Image data analysis

**FIGURE 2:**
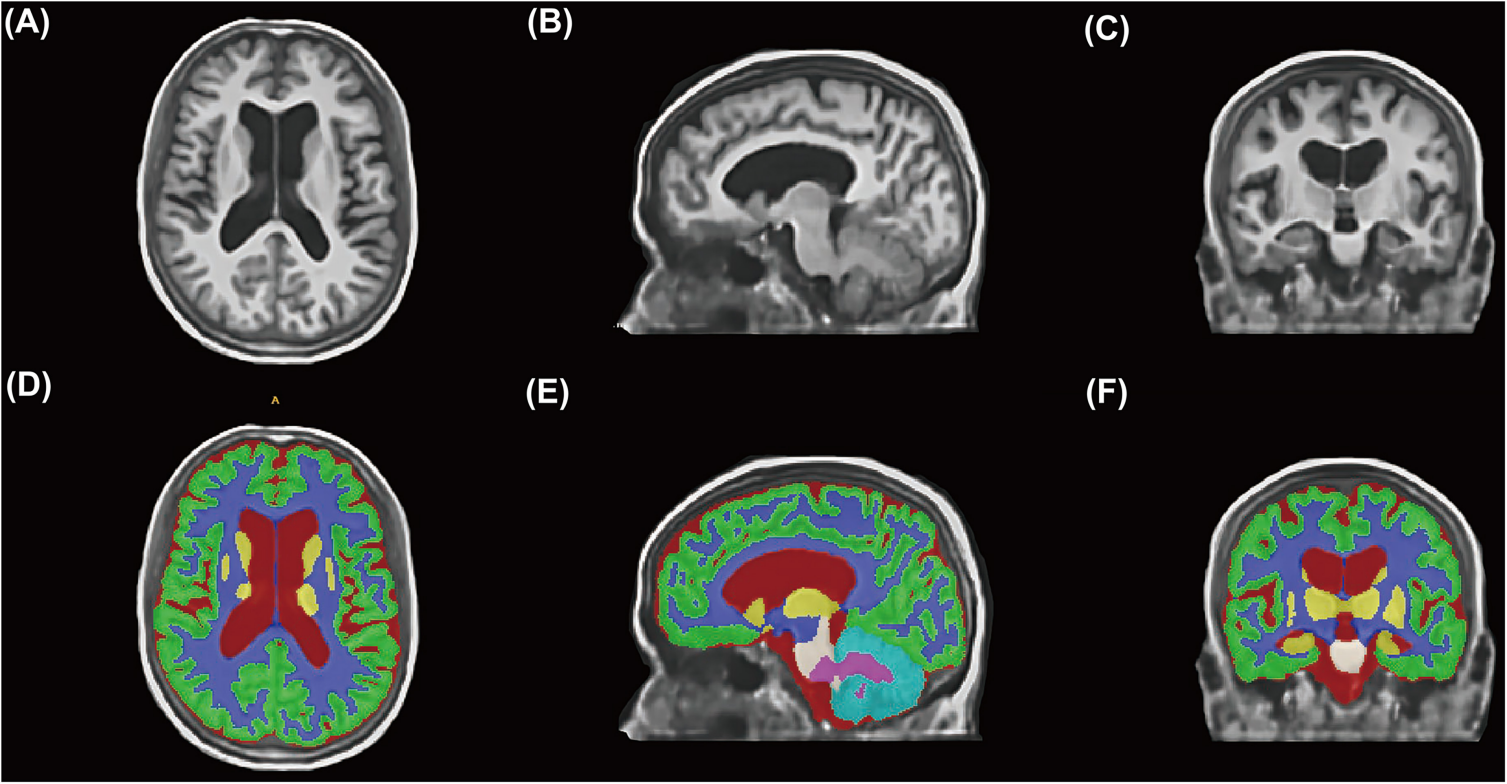
Automated brain tissue segmentation workflow using deep learning technology. **(A–C)** Representative clinical T1-weighted MRI images from a study participant displayed in axial **(A)**, sagittal **(B)**, and coronal **(C)** orientations after SynthSR reconstruction to standardized 1-mm isotropic resolution. **(D–F)** Corresponding automated brain tissue segmentation results generated by AssemblyNet deep learning algorithm. Color-coded regions represent different anatomical structures.

All image data analyses employed a standardized, fully automated processing pipeline to ensure objectivity and reproducibility. The workflow comprised the following steps: (1) Image reconstruction and standardization: Original 2D T1WI data were processed using the extensively validated deep learning model SynthSR (v1.0^2^) to reconstruct standardized MPRAGE-style images with 1-mm isotropic resolution, while automatically performing bias field correction and signal-to-noise ratio optimization. Image quality before and after processing was systematically evaluated by a neuroimaging expert (J. Lin). Data exhibiting significant motion artifacts, insufficient field of view resulting in incomplete skull coverage, or other severe image quality issues were excluded. (2) Automated brain tissue segmentation (Figure 2): Reconstructed T1WI images underwent fully automated brain tissue segmentation using the AssemblyNet deep learning tool^3^ within a Docker containerized environment. AssemblyNet represents an automated brain MRI analysis pipeline based on ensemble deep convolutional neural networks, incorporating a comprehensive workflow of preprocessing, segmentation, and post-processing: image denoising and bias field correction; affine registration to Montreal Neurological Institute standard space; tissue-specific signal intensity normalization; automated skull stripping; fine-grained brain tissue segmentation; and inverse registration to individual native space. AssemblyNet automatically generates quality ratings including Grade A (excellent, ready for use), Grade B (moderate, requiring manual verification), and Grade C (poor, unsuitable for use). All Grade C quality imaging data were excluded from this study. Additionally, two experienced specialists (neurologist Z. Xiao and neuroimaging expert J. Lin) performed dual manual verification of all segmentation results to ensure anatomical segmentation accuracy and consistency. Discrepant cases were resolved through consensus discussion.

### 2.5 Brain atrophy quantification

Based on AssemblyNet segmentation results, we calculated brain tissue volumetric parameters for each patient, including total gray matter volume, total white matter volume, total brain volume (sum of gray and white matter volumes), total cerebrospinal fluid volume (comprising lateral ventricles, third ventricle, fourth ventricle, and extracerebral cerebrospinal fluid spaces), and total intracranial volume (sum of total brain volume and cerebrospinal fluid volume). To eliminate inter-individual differences in head size and quantitatively assess brain atrophy severity, the following standardized volume fractions were calculated (Kawas et al., 2025): (1) gray matter fraction (GMF) = total gray matter volume/total intracranial volume; (2) white matter fraction (WMF) = total white matter volume/total intracranial volume; (3) brain parenchymal fraction (BPF) = (total gray matter volume + total white matter volume)/total intracranial volume; and (4) cerebrospinal fluid fraction (CSFF) = total cerebrospinal fluid volume/total intracranial volume. These fraction values range from 0 to 1, with lower values indicating greater atrophy severity for the corresponding tissue type; conversely, higher CSFF values indicate more severe brain atrophy.

### 2.6 Primary endpoint definition

Early neurological deterioration was defined as persistent neurological deterioration occurring during hospitalization, specifically characterized by an increase in NIHSS score of ≥2 points or an increase in motor function score of ≥1 point, with deterioration persisting for >24 h. Transient neurological fluctuations were excluded. For patients who experienced progressive symptom worsening prior to admission, those meeting the aforementioned NIHSS score change criteria were similarly classified as END events.

### 2.7 Statistical analysis

All statistical analyses were performed using R software (Version 4.2.2). The Shapiro-Wilk test was used to assess normality of continuous variables. Normally distributed continuous variables are presented as mean ± standard deviation and compared using independent samples *t*-tests; non-normally distributed continuous variables are presented as median (interquartile range) and compared using Mann-Whitney U tests. Categorical variables are presented as frequencies (percentages) and analyzed using Pearson chi-square tests or Fisher’s exact tests for between-group differences.

Prior to constructing multivariable logistic regression models, variance inflation factors (VIF) were calculated to assess multicollinearity among candidate variables. Variables with VIF ≥ 10 or tolerance ≤ 0.1 were excluded from multivariable analyses. To systematically evaluate independent associations between brain atrophy quantitative metrics (GMF, WMF, BPF, CSFF) and END, three progressively adjusted multivariable logistic regression models were constructed: Model 1 (crude model); Model 2 (partially adjusted model): adjusted for demographic and core clinical variables, including age, diabetes mellitus history, baseline mRS score, infarct lesion size, and involvement of key anatomical regions (lateral geniculate body, medial temporal lobe/hippocampal structures); Model 3 (fully adjusted model): further adjusted for laboratory parameters including neutrophil count, low-density lipoprotein cholesterol, fibrinogen, and uric acid levels, in addition to Model 2 variables. Restricted cubic spline (RCS) regression models were employed to evaluate dose-response relationships and potential non-linear associations between brain atrophy metrics and END risk. All statistical tests were two-sided, with *P* < 0.05 considered statistically significant.

## 3 Results

### 3.1 Patient characteristics and laboratory findings

This study included 206 patients with AChA territory infarction (Table 1), comprising 151 males (73.30%) with a mean age of 63 ± 13 years. Among these, 78 patients (37.86%) developed END. Patients in the END group were significantly older than those in the non-END group (*P* = 0.006) and had a higher prevalence of diabetes mellitus (*P* = 0.016). At admission, the END group demonstrated significantly higher mRS scores compared to the non-END group (*P* < 0.001). Imaging analysis revealed that the END group had a significantly higher proportion of large infarcts (≥20 mm) compared to the non-END group (*P* < 0.001). Regarding anatomical involvement, the END group showed significantly higher rates of lateral geniculate body (*P* = 0.020) and medial temporal lobe/hippocampal region (*P* = 0.028) involvement compared to the non-END group.

**TABLE 1:** Baseline characteristics of patients with anterior choroidal artery territory infarction stratified by early neurological progression.

Variables	Overall *N* = 206	Non-END *N* = 128	END *N* = 78	*P*-value
Age, years	63 ± 13	61 ± 14	66 ± 12	0.006
Sex, male	151 (73.30)	95 (74.22)	56 (71.79)	0.703
Current smoker	73 (35.44)	48 (37.50)	25 (32.05)	0.428
Alcohol consumption, *n* (%)	40 (19.42)	21 (16.41)	19 (24.36)	0.162
Baseline NIHSS score	3.0 (2.0, 6.0)	3.0 (2.0, 5.0)	4.0 (2.0, 7.0)	0.059
Baseline mRS score	2.0 (1.0, 4.0)	2.0 (1.0, 4.0)	3.0 (1.0, 4.0)	<0.001
**Medical history, *n* (%)**				
Hypertension	156 (75.73)	94 (73.44)	62 (79.49)	0.326
Diabetes mellitus	52 (25.24)	25 (19.53)	27 (34.62)	0.016
Hyperlipidemia	75 (36.41)	45 (35.16)	30 (38.46)	0.632
Atrial fibrillation	4 (1.94)	2 (1.56)	2 (2.56)	0.635
Previous stroke/TIA	37 (17.96)	24 (18.75)	13 (16.67)	0.706
Coronary artery disease	18 (8.74)	14 (10.94)	4 (5.13)	0.152
Large infarcts (≥20 mm), *n* (%)	41 (19.90)	16 (12.50)	25 (32.05)	<0.001
**Anatomical involvement, *n* (%)**				
Posterior limb of internal capsule	162 (78.64)	96 (75.00)	66 (84.62)	0.102
Corona radiata	152 (73.79)	91 (71.09)	61 (78.21)	0.260
Medial globus pallidus	14 (6.80)	8 (6.25)	6 (7.69)	0.690
Tail of caudate nucleus	23 (11.17)	11 (8.59)	12 (15.38)	0.133
Lateral thalamus	24 (11.65)	19 (14.84)	5 (6.41)	0.067
Lateral geniculate body	4 (1.94)	0 (0.00)	4 (5.13)	0.020
Medial temporal lobe/hippocampus	9 (4.37)	2 (1.56)	7 (8.97)	0.028
Intravenous thrombolysis, *n* (%)	38 (18.45)	27 (21.09)	11 (14.10)	0.210
**Antiplatelet therapy, *n* (%)**				**0.392**
No antiplatelet	5 (2.43)	4 (3.13)	1 (1.28)	–
Single antiplatelet	69 (33.50)	39 (30.47)	30 (38.46)	–
Dual antiplatelet	132 (64.08)	85 (66.41)	47 (60.26)	–

Values are presented as *n* (%), mean ± standard deviation, or median (interquartile range) as appropriate. Statistical comparisons performed using independent samples *t*-test for normally distributed continuous variables, Mann-Whitney U test for non-normally distributed continuous variables, and Pearson chi-square test or Fisher’s exact test for categorical variables. AChA, anterior choroidal artery; END, early neurological deterioration; NIHSS, National Institutes of Health Stroke Scale; mRS, modified Rankin Scale; TIA, transient ischemic attack.

Laboratory examination findings (Table 2) demonstrated that the END group had significantly higher neutrophil counts (*P* = 0.023), low-density lipoprotein cholesterol levels (*P* = 0.016), uric acid (*P* = 0.013), and fibrinogen levels (*P* = 0.019) compared to the non-END group. White blood cell count and fasting glucose levels showed an increasing trend in the END group (*P* = 0.053; *P* = 0.053), though these differences did not reach statistical significance.

**TABLE 2:** Laboratory parameters and brain volumetric measurements in patients with anterior choroidal artery territory infarction by early neurological deterioration status.

Variables	Overall *N* = 206	Non-END *N* = 128	END *N* = 78	*P*-value
White blood cell, × 10^9^/L	6.97 (5.87, 8.81)	6.86 (5.71, 8.68)	7.32 (6.31, 8.91)	0.053
Neutrophils, × 10^9^/L	4.71 (3.44, 6.19)	4.62 (3.34, 5.91)	4.89 (3.88, 6.67)	0.023
Lymphocytes, × 10^9^/L	1.68 (1.30, 2.08)	1.71 (1.38, 2.08)	1.64 (1.22, 2.24)	0.485
Monocytes, × 10^9^/L	0.42 (0.34, 0.56)	0.42 (0.34, 0.54)	0.43 (0.35, 0.57)	0.389
Red blood cells, × 10^12^/L	4.63 (4.33, 4.94)	4.63 (4.32, 4.93)	4.63 (4.42, 4.96)	0.665
Hemoglobin, g/L	143 (131, 151)	144 (135, 151)	142 (130, 152)	0.363
Platelets count, × 10^9^/L	231 (189, 268)	230 (189, 260)	231 (190, 269)	0.660
Total protein, g/L	69 (66, 74)	69 (66, 72)	72 (65, 77)	0.079
Albumin, g/L	41.1 ± 3.4	41.4 ± 3.1	40.7 ± 3.8	0.171
Alanine aminotransferase, U/L	18 (13, 25)	18 (13, 26)	17 (12, 23)	0.226
Aspartate aminotransferase, U/L	21 (17, 24)	20 (17, 25)	21 (17, 24)	0.956
Triglycerides, mmol/L	1.35 (0.97, 2.03)	1.36 (1.00, 2.12)	1.30 (0.94, 1.95)	0.423
Total cholesterol, mmol/L	5.09 ± 1.21	5.05 ± 1.11	5.15 ± 1.37	0.589
LDL cholesterol, mmol/L	3.21 (2.72, 3.78)	3.05 (2.65, 3.61)	3.27 (2.84, 3.98)	0.016
Creatinine, μmol/L	72 (63, 82)	72 (62, 82)	73 (63, 84)	0.486
Uric acid, μmol/L	331 (286, 387)	320 (267, 370)	347 (302, 414)	0.013
FBG, mmol/L	5.67 (5.08, 6.98)	5.53 (5.07, 6.83)	6.17 (5.13, 7.50)	0.053
Fibrinogen, g/L	3.03 (2.55, 3.55)	2.95 (2.44, 3.47)	3.23 (2.69, 3.71)	0.019
Total intracranial volume, cm^3^	1444 ± 128	1432 ± 128	1464 ± 126	0.085
White matter volume, cm^3^	442 ± 44	443 ± 46	441 ± 39	0.691
Gray matter volume, cm^3^	785 ± 71	782 ± 73	790 ± 68	0.423
Brain parenchyma volume, cm^3^	1227 ± 114	1225 ± 119	1231 ± 106	0.722
Cerebrospinal fluid volume, cm^3^	199 (177, 220)	186 (170, 209)	214 (195, 234)	<0.001
White matter fraction	0.306 ± 0.010	0.309 ± 0.010	0.301 ± 0.009	<0.001
Gray matter fraction	0.545 (0.536, 0.551)	0.547 (0.540, 0.553)	0.540 (0.534, 0.547)	<0.001
Brain parenchymal fraction	0.850 ± 0.019	0.855 ± 0.018	0.841 ± 0.017	<0.001
CSF fraction	0.139 ± 0.019	0.134 ± 0.018	0.148 ± 0.017	<0.001

Values are presented as median (interquartile range) or mean ± standard deviation as appropriate. Statistical comparisons performed using independent samples *t*-test for normally distributed variables and Mann-Whitney U test for non-normally distributed variables. Brain volumetric measurements obtained through automated segmentation using AssemblyNet following SynthSR image reconstruction. Volumetric fractions calculated as tissue volume divided by total intracranial volume. END, early neurological deterioration; FBG, fasting blood glucose; LDL, low-density lipoprotein; CSF, cerebrospinal fluid.

### 3.2 Brain atrophy quantification and volumetric analysis

**FIGURE 3:**
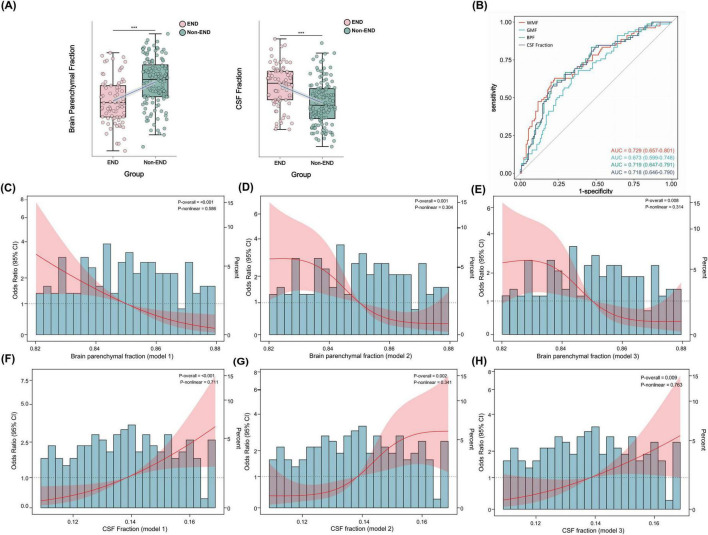
Brain atrophy quantification and early neurological deterioration risk assessment. **(A)** Comparison of standardized brain volumetric fractions (brain parenchymal fraction and cerebrospinal fluid fraction) between patients with early neurological deterioration (END) and those without (Non-END). ***P < 0.001. **(B)** Receiver operating characteristic (ROC) curves comparing discriminative performance of brain atrophy metrics for END prediction. **(C–H)** Restricted cubic spline analysis demonstrating dose-response relationships between brain atrophy metrics and END risk. **(C–E)** Show associations between brain parenchymal fraction and END; **(F–H)** show associations between cerebrospinal fluid fraction and END. Each metric is displayed across three progressive adjustment models: unadjusted **(C,F)**, partially adjusted **(D,G)**, and fully adjusted **(E,H)**.

Brain tissue segmentation analysis revealed no significant differences between groups in total intracranial volume, white matter volume, gray matter volume, or total brain parenchymal volume (all *P* > 0.05). However, significant differences emerged in standardized volume fraction analyses: the END group demonstrated significantly lower brain parenchymal fraction (*P* < 0.001), white matter fraction (*P* < 0.001), and gray matter fraction (*P* < 0.001) compared to the non-END group (Figure 3A). Additionally, the END group exhibited significantly larger cerebrospinal fluid volume (*P* < 0.001) and correspondingly elevated cerebrospinal fluid fraction (*P* < 0.001).

### 3.3 Multivariable logistic regression analysis

To evaluate independent associations between brain atrophy quantitative metrics and END risk, three progressively adjusted multivariable logistic regression models were constructed (Table 3). In the fully adjusted model (Model 3), each 0.01-unit increase in WMF was associated with a 58% reduction in END risk (OR = 0.42, 95% CI: 0.26–0.65, *P* < 0.001). Per standard deviation increase in WMF corresponded to a 59% reduction in END risk (OR = 0.41, 95% CI: 0.25–0.65, *P* < 0.001). Similarly, BPF demonstrated a significant inverse association with END risk: each 0.01-unit increase was associated with a 32% reduction in END risk (OR = 0.68, 95% CI: 0.53–0.87, *P* = 0.002), while each standard deviation increase corresponded to a 51% risk reduction (OR = 0.49, 95% CI: 0.31–0.77, *P* = 0.002).

**TABLE 3:** Multivariable logistic regression analysis of brain atrophy metrics as predictors of early neurological deterioration in anterior choroidal artery territory infarction.

Variables	Model 1		Model 2		Model 3	
	OR (95% CI)	*P*-value	OR (95% CI)	*P*-value	OR (95% CI)	*P*-value
GMF (per 0.01 units)	0.54 (0.39, 0.72)	<0.001	0.64 (0.44, 0.92)	0.016	0.72 (0.48, 1.06)	0.095
**GMF**						
Quartile 1	1.00 (ref)	–	1.00 (ref)	–	1.00 (ref)	–
Quartile 2	0.6 (0.27, 1.31)	0.202	0.66 (0.28, 1.53)	0.333	0.65 (0.27, 1.57)	0.342
Quartile 3	0.40 (0.18, 0.88)	0.024	0.36 (0.14, 0.90)	0.032	0.40 (0.15, 1.05)	0.065
Quartile 4	0.11 (0.04, 0.29)	<0.001	0.20 (0.06, 0.59)	0.005	0.25 (0.07, 0.79)	0.022
P for trend	–	<0.001	–	0.002	0.72 (0.48, 1.06)	0.013
WMF (per 0.01 units)	0.41 (0.29, 0.57)	<0.001	0.40 (0.25, 0.61)	<0.001	0.42 (0.26, 0.65)	<0.001
WMF (per SD increase)	0.40 (0.28, 0.56)	<0.001	0.39 (0.24, 0.61)	<0.001	0.41 (0.25, 0.65)	<0.001
**WMF**						
Quartile 1	1.00 (ref)	–	1.00 (ref)	–	1.00 (ref)	–
Quartile 2	0.20 (0.09, 0.46)	<0.001	0.25 (0.10, 0.61)	0.003	0.22 (0.08, 0.56)	0.002
Quartile 3	0.17 (0.07, 0.39)	<0.001	0.19 (0.06, 0.51)	0.001	0.21 (0.07, 0.59)	0.004
Quartile 4	0.08 (0.03, 0.21)	<0.001	0.09 (0.03, 0.29)	<0.001	0.09 (0.02, 0.29)	<0.001
P for trend	–	<0.001	–	<0.001	0.22 (0.08, 0.56)	<0.001
BPF (per 0.01 units)	0.63 (0.53–0.76)	<0.001	0.65 (0.51–0.82)	<0.001	0.68 (0.53–0.87)	0.002
BPF (per SD increase)	0.43 (0.30–0.59)	<0.001	0.45 (0.29–0.69)	<0.001	0.49 (0.31–0.77)	0.002
**BPF**						
Quartile 1	1.00 (ref)	–	1.00 (ref)	–	1.00 (ref)	–
Quartile 2	0.47 (0.21–1.03)	0.063	0.63 (0.27–1.49)	0.294	0.60 (0.24–1.45)	0.256
Quartile 3	0.24 (0.10–0.53)	<0.001	0.21 (0.08–0.56)	0.002	0.25 (0.09–0.69)	0.008
Quartile 4	0.15 (0.06–0.35)	<0.001	0.22 (0.07–0.65)	0.007	0.26 (0.08–0.83)	0.024
P for trend	–	<0.001	–	0.001	–	0.008
CSF fraction (per 0.01 units)	1.56 (1.32–1.88)	<0.001	1.51 (1.22–1.91)	<0.001	1.52 (1.20–1.94)	<0.001
CSF fraction (per SD increase)	2.31 (1.68–3.27)	<0.001	2.19 (1.45–3.40)	<0.001	2.19 (1.41–3.50)	<0.001
**CSF fraction**						
Quartile 1	1.00 (ref)	–	1.00 (ref)	–	1.00 (ref)	–
Quartile 2	1.44 (0.57–3.73)	0.447	0.96 (0.32–2.83)	0.935	1.00 (0.34–2.98)	0.994
Quartile 3	3.19 (1.34–7.98)	0.01	3.13 (1.13–9.13)	0.031	2.82 (0.97–8.58)	0.061
Quartile 4	7.29 (3.08–18.52)	<0.001	5.59 (1.90–17.42)	0.002	6.22 (1.98–20.84)	0.002
P for trend	–	<0.001	–	<0.001	–	<0.001

Values are expressed as odds ratios (ORs) with 95% confidence intervals (CIs). BPF, brain parenchymal fraction; CI, confidence interval; CSF, cerebrospinal fluid; GMF, gray matter fraction; OR, odds ratio; Ref, reference category; SD, standard deviation; WMF, white matter fraction.

Conversely, CSFF showed a significant positive association with END risk. In the fully adjusted model, each 0.01-unit increase in CSFF was associated with a 52% increase in END risk (OR = 1.52, 95% CI: 1.20–1.94, *P* < 0.001), while each standard deviation increase corresponded to a 119% increase in END risk (OR = 2.19, 95% CI: 1.41–3.50, *P* < 0.001). Quartile analysis of CSFF demonstrated an escalating trend in END risk with increasing CSFF values. Using the lowest quartile as reference, the adjusted ORs for Q3 and Q4 were 2.82 (95% CI: 0.97–8.58, *P* = 0.061) and 6.22 (95% CI: 1.98–20.84, *P* = 0.002), respectively. Notably, GMF lost statistical significance in its association with END in the fully adjusted model (OR = 0.72, 95% CI: 0.48–1.06, *P* = 0.095).

### 3.4 Receiver operating characteristic analysis and predictive performance

Receiver operating characteristic (ROC) curve analysis (Figure 3B and Supplementary Table 1) demonstrated that white matter fraction possessed the highest discriminative ability, with an AUC of 0.73 (95% CI: 0.66–0.80) and an optimal cutoff value of 0.302. Brain parenchymal fraction and cerebrospinal fluid fraction showed comparable predictive performance, both achieving AUCs of 0.72 (BPF 95% CI: 0.65–0.79; CSFF 95% CI: 0.65–0.79), with optimal cutoff values of 0.844 and 0.147, respectively. Gray matter fraction demonstrated relatively lower predictive capability, with an AUC of 0.67 (95% CI: 0.60–0.75).

Regarding diagnostic performance, cerebrospinal fluid fraction exhibited the best overall accuracy (71%), sensitivity (79%), and specificity (58%). In contrast, other brain atrophy metrics, while possessing moderate discriminative ability (AUC > 0.65), demonstrated lower overall accuracy (28%–36%).

### 3.5 Non-linear association analysis

To further explore association patterns between brain atrophy metrics and END, we employed RCS analysis to evaluate potential non-linear relationships (Figures 3C–H). Analysis results consistently demonstrated that brain atrophy indicators exhibited significant linear association patterns with END. For brain parenchymal fraction, the overall association *p*-values were <0.001, <0.001, and 0.008 in unadjusted, partially adjusted, and fully adjusted models, respectively, while non-linearity tests yielded *p*-values of 0.586, 0.314, and 0.314, respectively, none reaching statistical significance. Cerebrospinal fluid fraction associations with END risk demonstrated a converse pattern, similarly showing significant overall associations (*P*-overall < 0.01 across all three models) without significant non-linear characteristics (both *P*-non-linear > 0.05).

## 4 Discussion

This study represents the first investigation to demonstrate associations between quantitative brain atrophy metrics (WMF, BPF, and CSFF) and END in patients with AChA territory infarction based on routine clinical MRI. Our findings indicate that in fully adjusted models, each 0.01-unit increase in WMF and BPF was associated with 58% and 32% reductions in END risk, respectively, while each 0.01-unit increase in CSFF corresponded to a 52% increase in END risk. Furthermore, ROC curve analysis provides robust evidence supporting CSFF as having optimal predictive capability (AUC = 0.72), with high overall accuracy (71%), sensitivity (79%), and specificity (58%), further emphasizing its clinical utility in quantifying END risk. These findings establish automated brain atrophy measurement as a clinically actionable biomarker capable of significantly enhancing risk stratification in this high-risk stroke population.

Accumulating evidence demonstrates the important value of brain atrophy in predicting post-stroke functional outcomes. A meta-analysis published by Monteiro and colleagues in 2022 encompassed multiple studies and revealed that despite heterogeneous neuroimaging methods for assessing brain atrophy—including brain atrophy indices, automated cerebrospinal fluid volume measurements, global cortical atrophy scales, and Standards for Reporting Vascular Changes on Neuroimaging—all studies consistently demonstrated that brain atrophy serves as an independent predictor of poor 3-month functional outcomes in acute ischemic stroke patients following mechanical thrombectomy (Monteiro et al., 2022). Subsequently Brugnara et al. (2024) further validated these findings. Their study included 1,103 ischemic stroke patients with middle cerebral artery territory occlusion who underwent endovascular treatment, employing visual assessment of age-related white matter changes (ARWMC) and brain atrophy markers from non-contrast CT data. They similarly confirmed that both ARWMC and cortical atrophy were independent predictors of 90-day clinical outcomes. Notably, incorporation of brain atrophy indices enhanced machine learning model prediction accuracy, with atrophy indicators ranking among the top five predictive factors. However, existing research has predominantly focused on large vessel occlusion strokes, while investigations targeting this specific stroke subtype of small vessel territory infarctions such as AChA, particularly regarding prediction of high-risk events like END, remain absent. Our study provides the first evidence confirming independent associations between quantitative brain atrophy metrics and END in AChA territory infarction, offering novel neuroimaging biomarkers for risk prediction in this high-risk stroke subtype.

The association between brain atrophy and increased END risk in patients with AChA territory infarction can be reasonably explained through multiple pathophysiological mechanisms. First, brain reserve refers to the brain’s capacity to maintain normal function or compensate for functional deficits when confronted with pathological injury. This concept may be attributed to various mechanisms, including neuroplasticity, neurogenesis, cortical structural integrity, and synaptic density (Wardlaw et al., 2013; Park et al., 2024). Brain atrophy, as a core imaging biomarker of diminished brain reserve capacity, demonstrates severity that inversely correlates with an individual’s resistance to acute cerebrovascular events and capacity for post-injury functional reorganization (Cheng et al., 2025; Pedraza et al., 2020). Therefore, patients with significant brain atrophy possess relatively vulnerable neurological compensatory mechanisms when experiencing acute ischemia in the AChA territory, rendering them more susceptible to persistent functional deterioration. Second, brain atrophy exhibits complex bidirectional associations with multiple cardiovascular and cerebrovascular risk factors. During physiological aging, brain volume demonstrates progressive reduction, a phenomenon particularly pronounced after age 60. Concurrently, aging is commonly accompanied by increased cardiovascular comorbidity burden, including hypertension, diabetes mellitus, and atherosclerosis, which further accelerate brain tissue atrophy through pathways involving chronic ischemia, oxidative stress, and inflammatory responses (Wang et al., 2025). The cumulative effects of these risk factors promote the development and progression of cerebral small vessel disease, which may manifest clinically as subclinical cognitive decline, increased functional dependency, or acute lacunar stroke syndromes. Therefore, radiologically detected brain atrophy represents not merely localized brain tissue volume reduction, but rather a comprehensive reflection of systemic vulnerability.

Our findings possess considerable clinical translational value and practical guidance implications. We successfully leveraged powerful convolutional neural network tools (SynthSR and AssemblyNet) to achieve effective bridging between real-world clinical practice and scientific research, enabling comprehensive utilization and in-depth analysis of vast heterogeneous imaging datasets generated in clinical settings. Although certain limitations may exist regarding precise volumetric measurements, our results confirm that fully automated brain atrophy quantification based on clinical MRI remains a robust predictor of END. This technological framework demonstrates excellent generalizability, allowing different regions and institutions to construct personalized prediction standards based on their own datasets and integrate these into current multimodal medical data analysis systems. Furthermore, in current clinical practice, aggressive antiplatelet strategies such as tirofiban are often initiated only as “rescue therapy” following END occurrence rather than as preventive measures, due to bleeding risk considerations and lack of effective risk prediction tools. The quantitative brain atrophy assessment provided by our study can serve as an important adjunctive predictive factor, accurately identifying high-risk END patients within the critical 72-h time window and enabling early risk stratification, thereby providing crucial clinical decision-making support for implementing individualized treatment strategies and optimizing benefit-risk ratios.

Several limitations warrant acknowledgment. First, the retrospective design precludes causal inference and introduces potential for selection and information bias. Second, as this study was conducted exclusively in two stroke centers in China, the generalizability of our findings to other ethnic populations and healthcare settings remains uncertain. Third, the male predominance in our cohort may limit the applicability of these findings to female patients. While this sex distribution reflects the true epidemiological characteristics of AChA territory infarction given our consecutive enrollment approach, future studies should incorporate more balanced sex ratios to explore potential sex-specific associations. Fourth, the two participating centers utilized MRI scanners with different field strengths. Although we employed SynthSR technology to reconstruct all images into standardized 1-mm isotropic MPRAGE-style images and verified measurement consistency across scanners, residual measurement bias cannot be entirely excluded. Accordingly, we prudently limited our analyses to global brain tissue compartments (gray matter, white matter, and cerebrospinal fluid) rather than pursuing fine-grained regional analyses that are more susceptible to scanner-related variability. Fifth, while brain atrophy metrics demonstrated significant associations in multivariable models, their discriminative ability when used in isolation was modest. It is important to emphasize that our study objective was to establish independent associations between brain atrophy and END, rather than to develop a standalone prediction tool. Single imaging biomarkers typically exhibit limited predictive performance; their clinical utility lies in integration into comprehensive, multivariable risk assessment models. Sixth, despite extensive covariate adjustment, residual confounding from unmeasured factors—including treatment heterogeneity and other cerebral small vessel disease markers—cannot be ruled out. Finally, this investigation assessed only short-term neurological deterioration during hospitalization and did not capture long-term functional outcomes. Future large-scale, prospective, multicenter studies employing standardized scanning protocols, enrolling diverse populations with balanced sex distribution, and incorporating extended follow-up periods are needed to validate and extend these findings.

## 5 Conclusion

Our findings demonstrate that automated brain atrophy quantification of routine clinical MRI using deep learning technology can effectively predict END risk in patients with AChA territory infarction. WMF, BPF, and CSFF, as objective indicators of brain reserve capacity, maintained significant independent predictive value after multivariable adjustment. This study provides clinicians with a risk assessment tool based on routine imaging examinations, facilitating identification of high-risk patients during the acute phase, guiding individualized treatment strategy development, and potentially improving clinical management and patient outcomes in this specific stroke subtype.

## Data Availability

The datasets presented in this article are not readily available because the clinical datasets used and/or analyzed during the current study are available from the corresponding author upon reasonable request. However, the original imaging data (including raw DICOM files and processed imaging datasets) are not available for sharing due to patient privacy protection requirements and institutional data management policies. Requests to access the datasets should be directed to Aihuan Zhang, zgaihuan@163.com.

## Footnotes

1
https://stroke-maps.github.io/anterior-choroidal

2
https://github.com/BBillot/SynthSR

3
https://github.com/volBrain/AssemblyNet
