# Supplementary material for: Automated brain atrophy quantification from clinical MRI predicts early neurological deterioration in anterior choroidal artery territory infarction
Source: Front Neurosci. 2025 Dec 18;19:1714159. doi: 10.3389/fnins.2025.1714159 (PMC12756492; doi:10.3389/fnins.2025.1714159)
Supplement: Supplementary file 1 [file Table_1.docx]

| **Supplementary Table S1.** Receiver Operating Characteristic Analysis of Brain Atrophy Metrics for Predicting Early Neurological Deterioration in Anterior Choroidal Artery Territory Infarction | | | | | |
| --- | --- | --- | --- | --- | --- |
| **NAR Parameter** | **AUC**  **(95% CI)** | **Accuracy**  **(95% CI)** | **Sensitivity**  **(95% CI)** | **Specificity**  **(95% CI)** | **Cut-off Value** |
| WMF | 0.73 (0.66-0.80) | 0.28 (0.22-0.35) | 0.22 (0.15 - 0.29) | 0.38 (0.28 - 0.49) | 0.302 |
| GMF | 0.67 (0.60-0.75) | 0.36 (0.30-0.43) | 0.38 (0.29 - 0.46) | 0.35 (0.24 - 0.45) | 0.544 |
| BPF | 0.72 (0.65-0.79) | 0.30 (0.23-0.36) | 0.24 (0.17 - 0.32) | 0.38 (0.28 - 0.49) | 0.844 |
| CSF Fraction | 0.72 (0.65-0.79) | 0.71 (0.64-0.77) | 0.79 (0.72 - 0.86) | 0.58 (0.47 - 0.69) | 0.147 |
| Receiver operating characteristic (ROC) curves were constructed to evaluate the discriminative performance of brain atrophy metrics for early neurological deterioration prediction. Cut-off values were determined using the Youden index method (maximum sensitivity + specificity - 1). Performance metrics presented with 95% confidence intervals calculated using DeLong's method for AUC comparisons and Wilson score intervals for sensitivity and specificity estimates. AUC = area under the curve; BPF = brain parenchymal fraction; CI = confidence interval; CSF = cerebrospinal fluid; GMF = gray matter fraction; WMF = white matter fraction. | | | | | |
